# Supplementary material for: Molecular profiling of aromatase inhibitor sensitive and resistant ER+HER2- postmenopausal breast cancers
Source: Nat Commun. 2023 Jul 7;14:4017. doi: 10.1038/s41467-023-39613-z (PMC10328947; doi:10.1038/s41467-023-39613-z)
Supplement: Supplementary file 1 — Supplementary Information [file 41467_2023_39613_MOESM1_ESM.pdf]

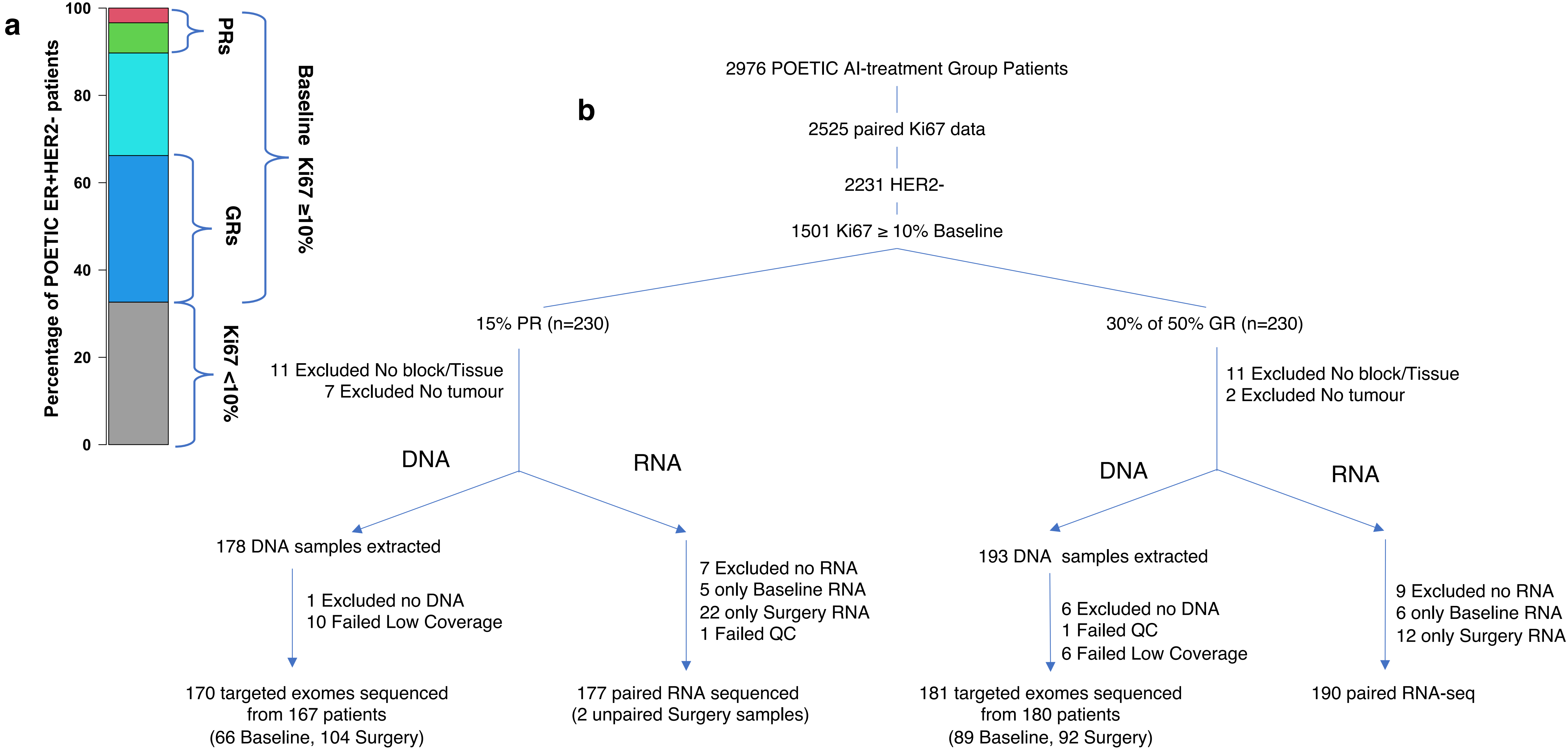

Supplementary Figure 1. **Sample selection. a** Barplot showing estimate of the percentage of GRs and PRs from POETIC AI-treated group. Source data are provided as a Source Data file. **b** Consort Diagram.

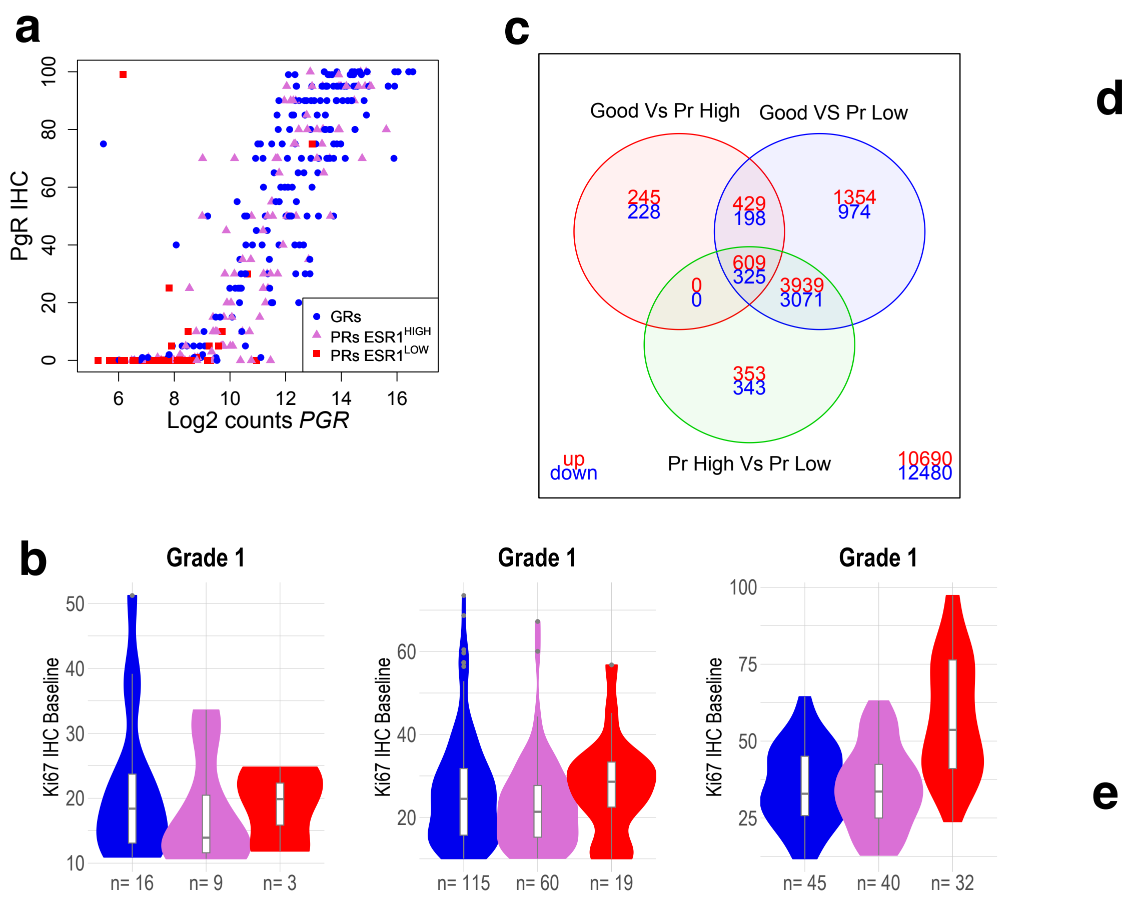

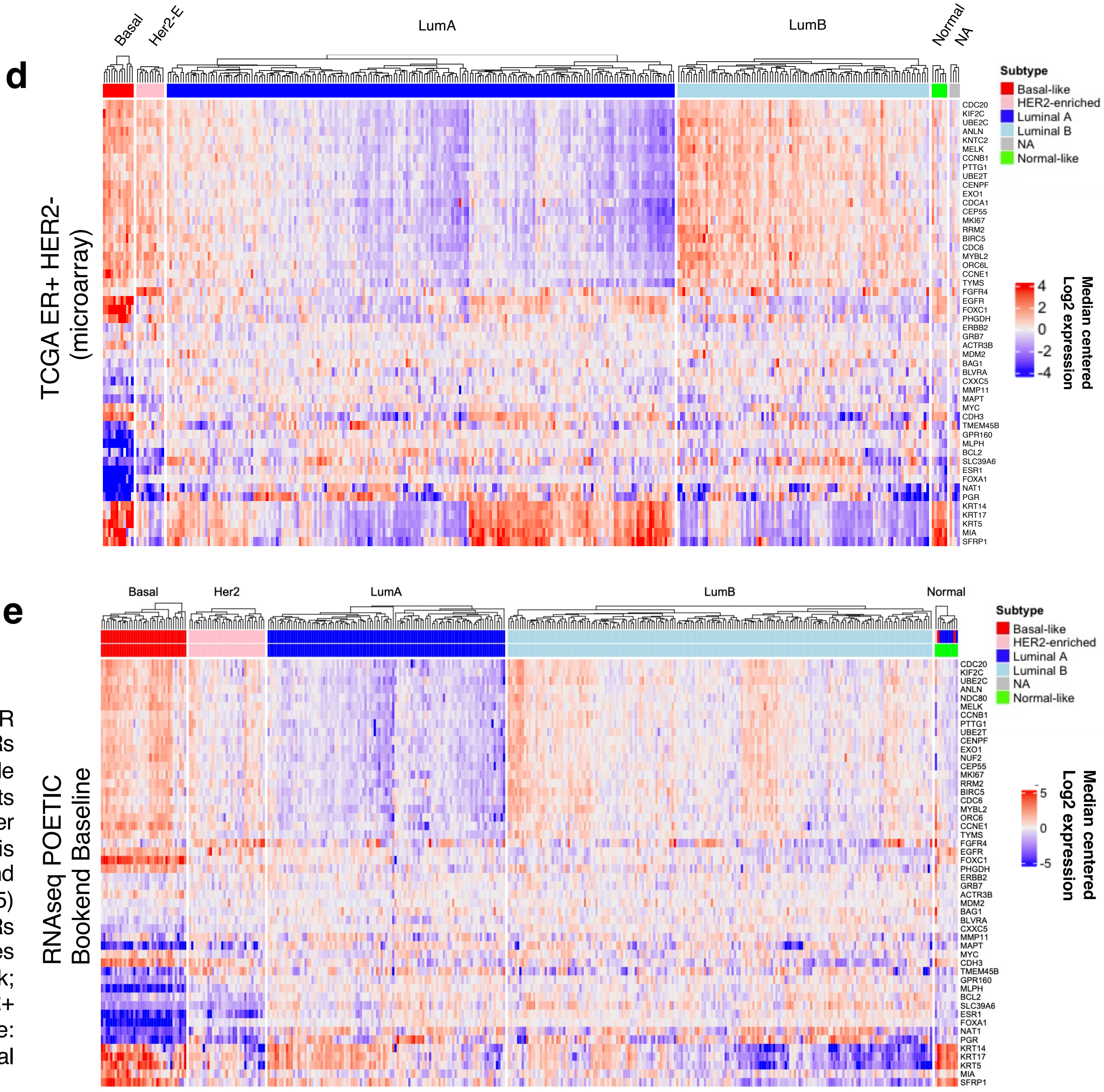

Supplementary Figure 2. **Differences in expression and grade. a** Scatter plot of PGR gene expression and PgR IHC% (GRs [blue dot], PRs ESR1^{HIGH} [orcid triangle] and PRs ESR1^{LOW} [red square]). **b** Violin/Boxplot of baseline Ki67 IHC values for tumor grade separated by GRs (blue), PRs ESR1^{HIGH} (orcid) and PRs ESR1^{LOW} (red). Boxplots present 25th , 50th (median), and 75th percentile values. Whiskers extend no larger than ±1.5 times the inter-quartile range with outliers plotted individually beyond this range. The number of independent samples used for comparisons between GRs and PRs is shown. **c** Venn diagram of overlap between up/down significantly (FDR < 0.05) differentially expressed genes between GRs vs all PRs, GRs vs PRs ESR1^{LOW} and GRs vs PRs ESR1^{HIGH}. Heatmaps of median centered log2 gene expression values supervised by PAM50 subtypes (LumA=blue; LumB=lightblue; HER2-enriched=pink; Basal=red; Normal=green) of PAM50-subtyping genes (log2 FC from median) ER+ HER2- TCGA BC microarray (**d**) and for POETIC RNAseq (**e**) data from this study. Note: POETIC RNAseq data includes subtype categories including and excluding the Normal subtype. Source data are provided as a Source Data file.

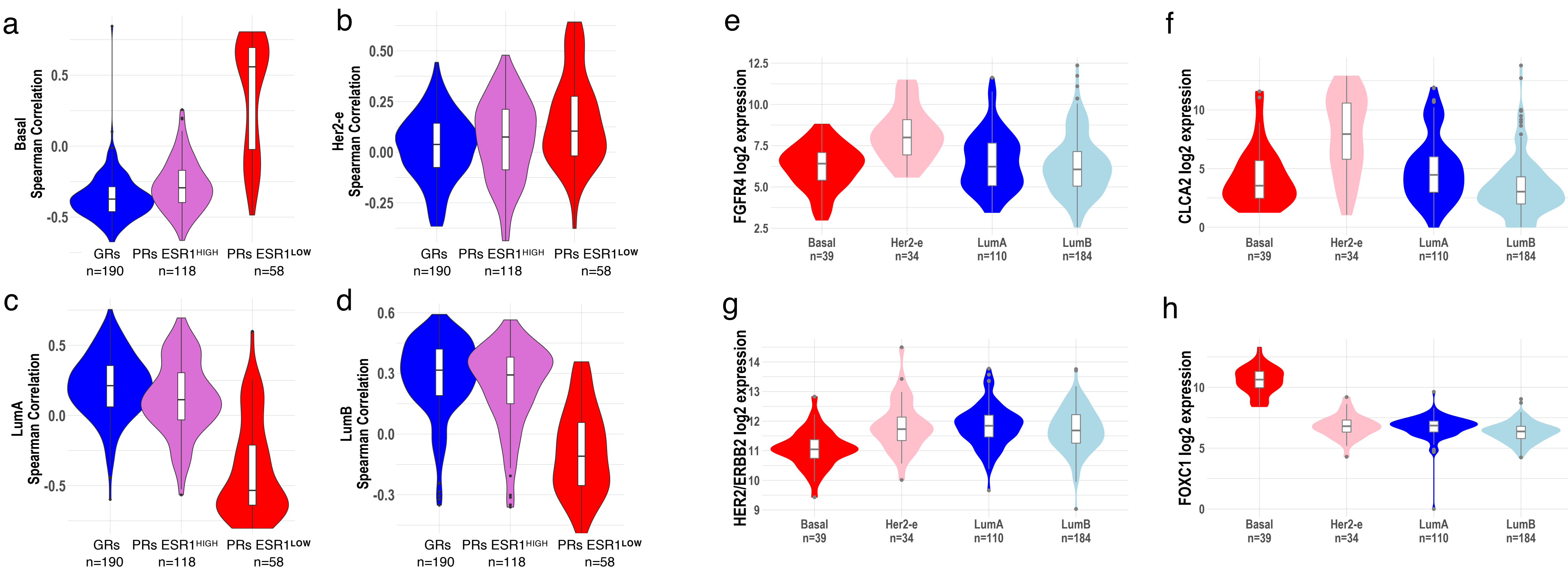

Supplementary Figure 3**. Subtype correlations and gene expression.** Violin/Boxplot of Spearman correlations for Basal (**a**), Her-e (**b**), LumA (**c**) and LumB (**d**) for GRs (blue), PRs ESR1^{HIGH} (green) and PRs ESR1^{LOW} (red). Boxplot of *FGFR4* (**e**), *CLCA2* (**f**), HER2/ERBB2 (**g**) and FOXC1 (**h**) expression for each intrinsic subtype. Boxplots present 25th , 50th (median), and 75th percentile values. Whiskers extend no larger than ±1.5 times the inter-quartile range with outliers plotted individually beyond this range. The number of independent samples used for comparisons between GRs and PRs is shown. Source data are provided as a Source Data file.

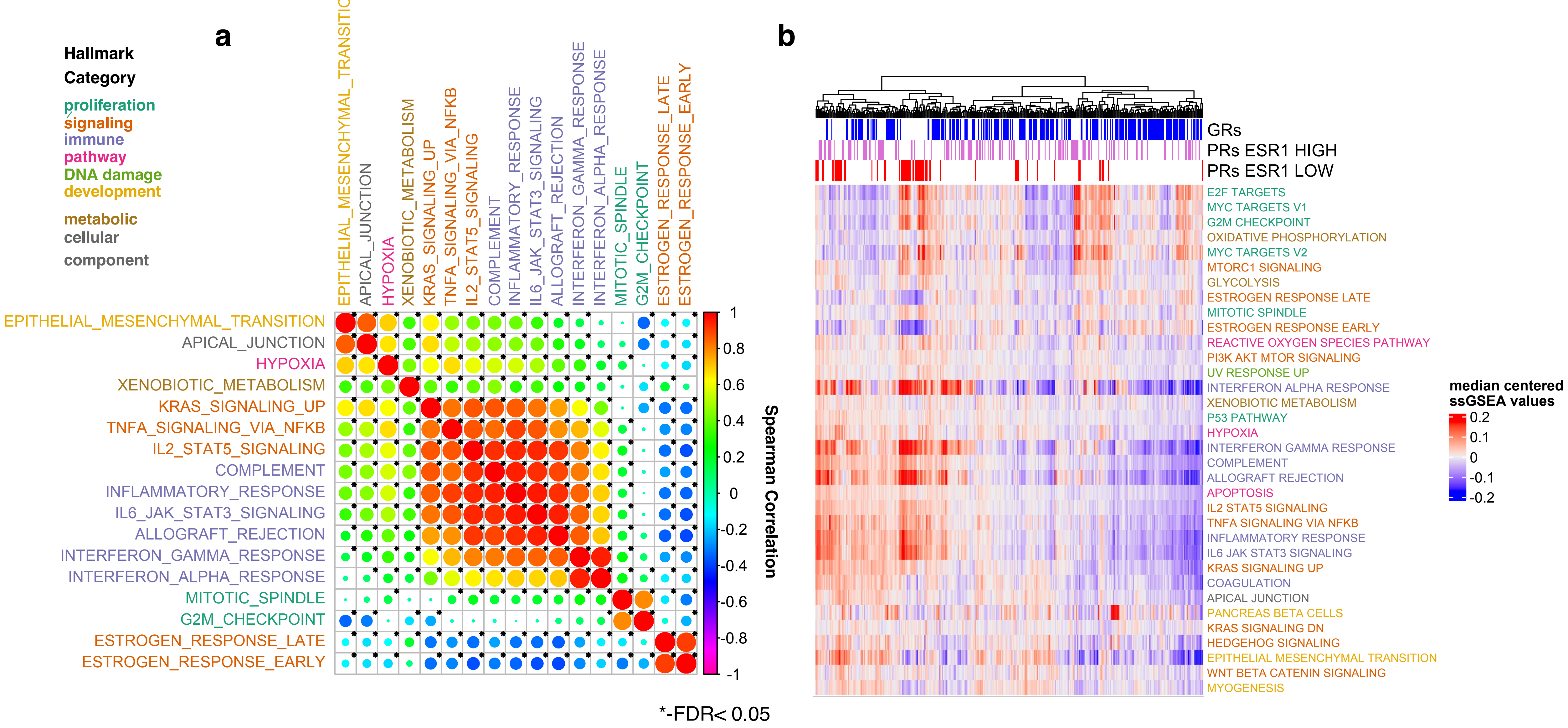

Supplementary Figure 4. **Process correlations and heatmap**. **a** Plot of single sample Gene Set Enrichment Analysis (ssGSEA) score Spearman correlations between MSD Hallmark gene sets for GRs and all PRs. Hallmarks with FDR < 0.05 in any comparison in Figure 3a shown. **b** Heatmap of median centered ssGSEA scores for these hallmarks. Hallmarks are colored by biological process category. Source data are provided as a Source Data file.

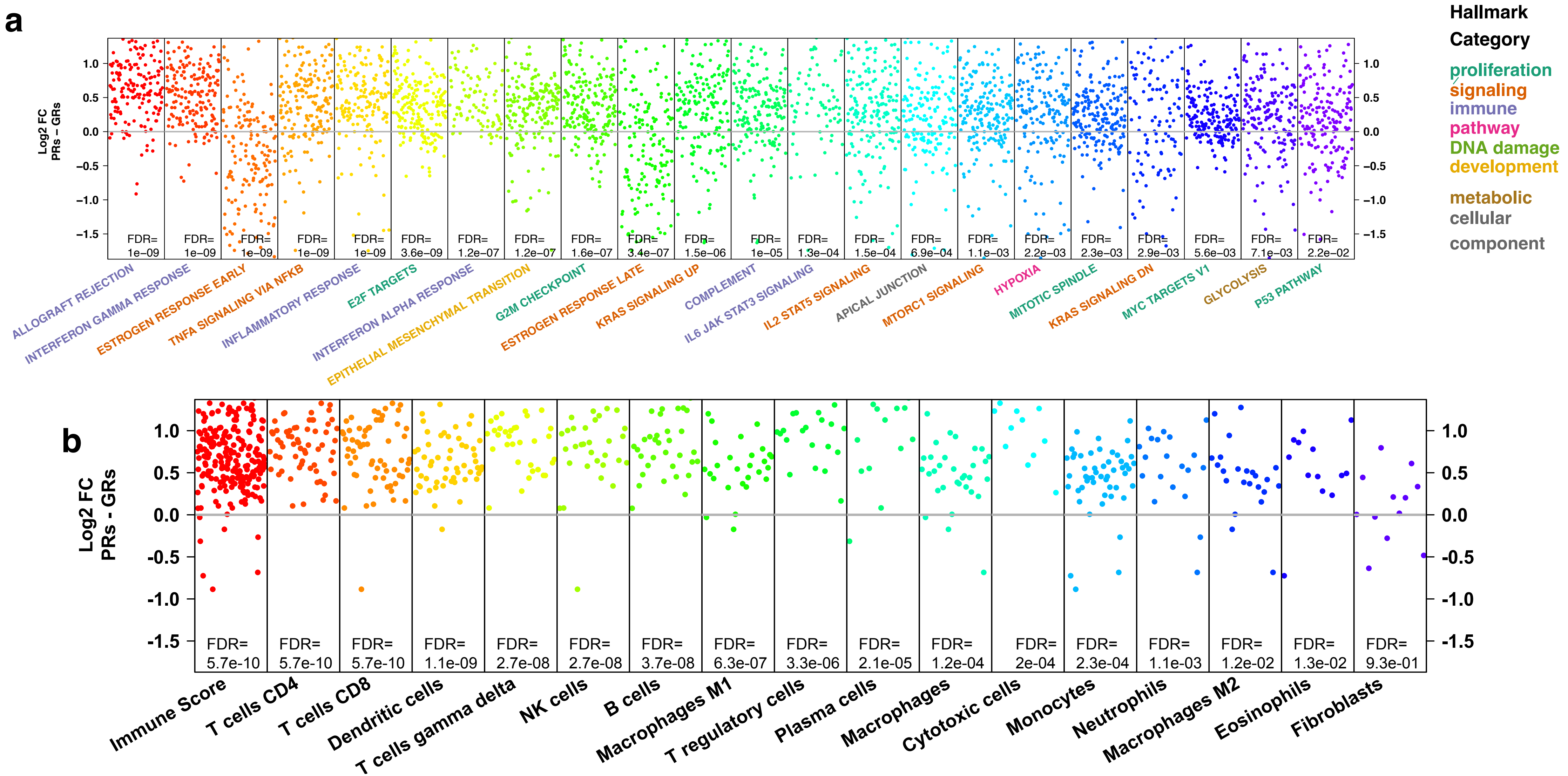

Supplementary Figure 5. **GSEA and TME gene expression for PRs ESR1^{LOW}.** Plot of log2 FC PRs ESR1^{Low} – GRs for individual genes within MSD Hallmark gene sets that are significantly different between PRs ESR1^{Low} and GRs in Gene Set Enrichment Analysis (GSEA) (**a**) and all Consensus Tumor Microenvironment (TME) BC gene sets (**b**). FDR values from GSEA analysis. Hallmarks are colored by biological process category. Source data are provided as a Source Data file.

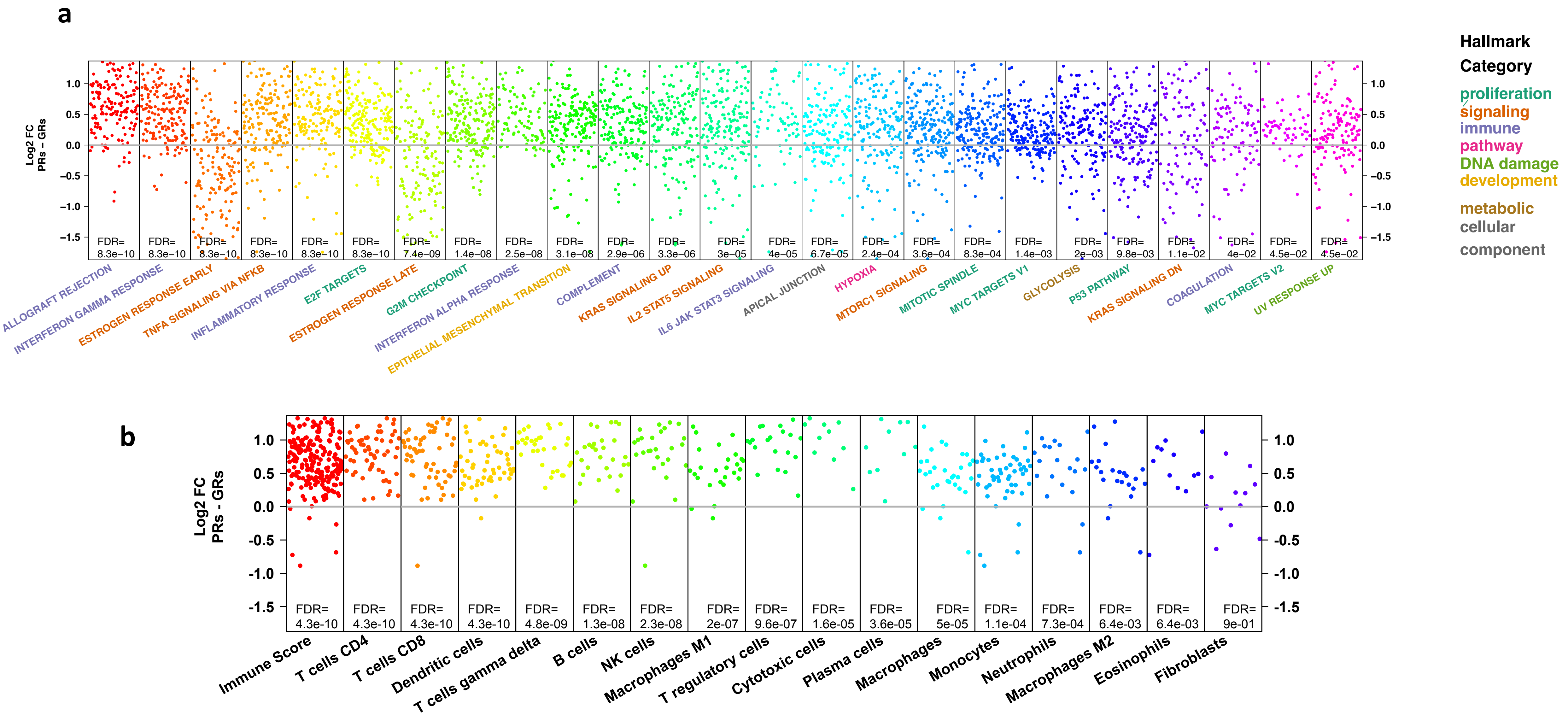

Supplementary Figure 6**. GSEA and TME gene expression for PRs.** Plot of log2 FC PRs – GRs for individual genes within MSD Hallmark gene sets that are significantly different between PRs and GRs in Gene Set Enrichment Analysis (GSEA) (**a**) and all Consensus Tumor Microenvironment (TME) BC gene sets (**b**). FDR values from GSEA analysis. Hallmarks are colored by biological process category. Source data are provided as a Source Data file.

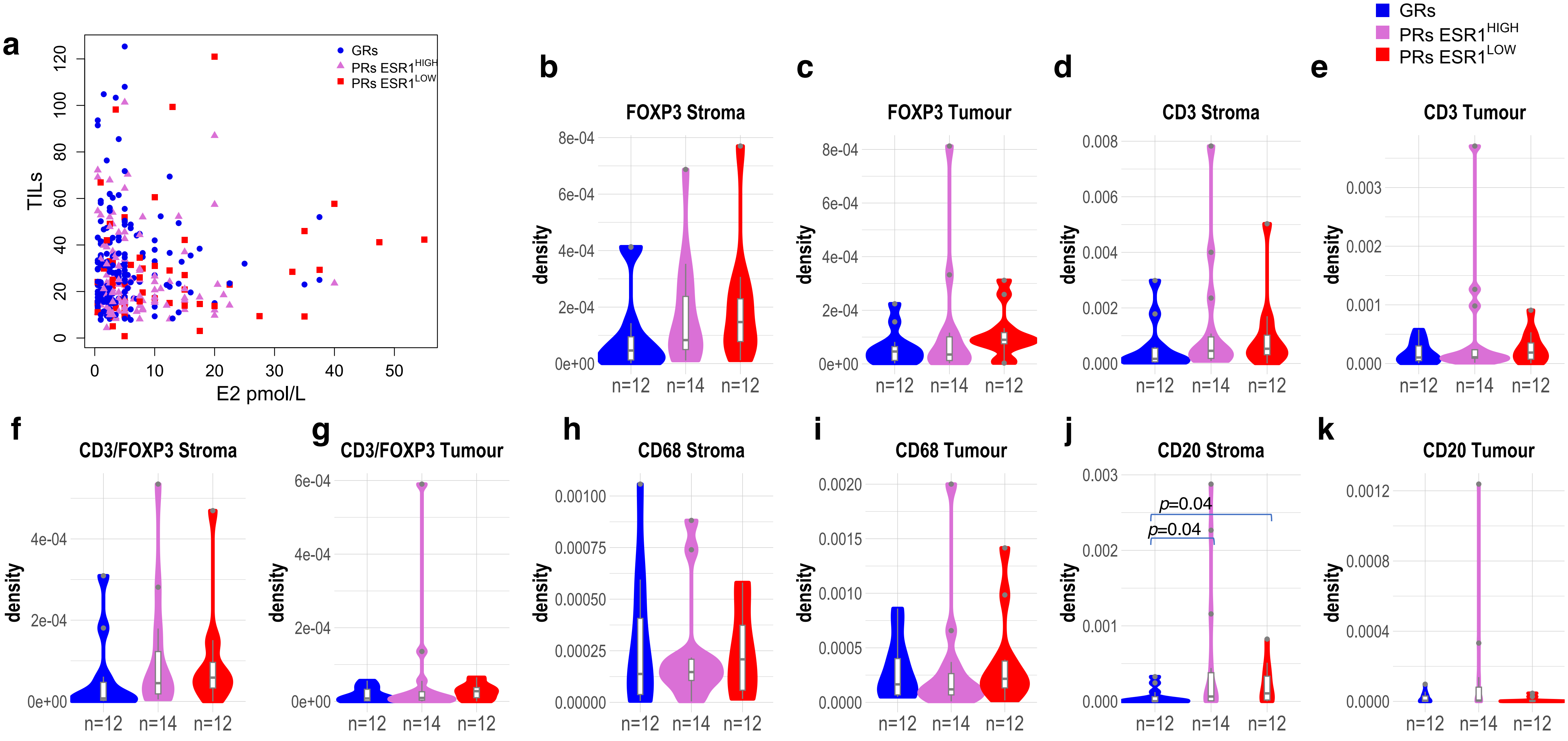

Supplementary Figure 7. **Immune markers. a** Scatter plot of TILs and estradiol (E2 pmol/L) (GRs [blue dot], PRs ESR1HIGH [orcid triangle] and PRs ESR1LOW [red square]). Boxplots of density of immune markers for stroma and tumor compartments for FOXP3 (**b-c**), CD3 (**d-e**), cells positive for both CD3 and FOXP3(**f-g**), CD68(**h-i**) and CD20 (**j-k**). Boxplots present 25th , 50th (median), and 75th percentile values. Whiskers extend no larger than ±1.5 times the inter-quartile range with outliers plotted individually beyond this range. The number of independent samples used for comparisons between GRs and PRs is shown. Significant differences (p<0.05) determined by two-sided Mann-Whitney tests. Source data are provided as a Source Data file.

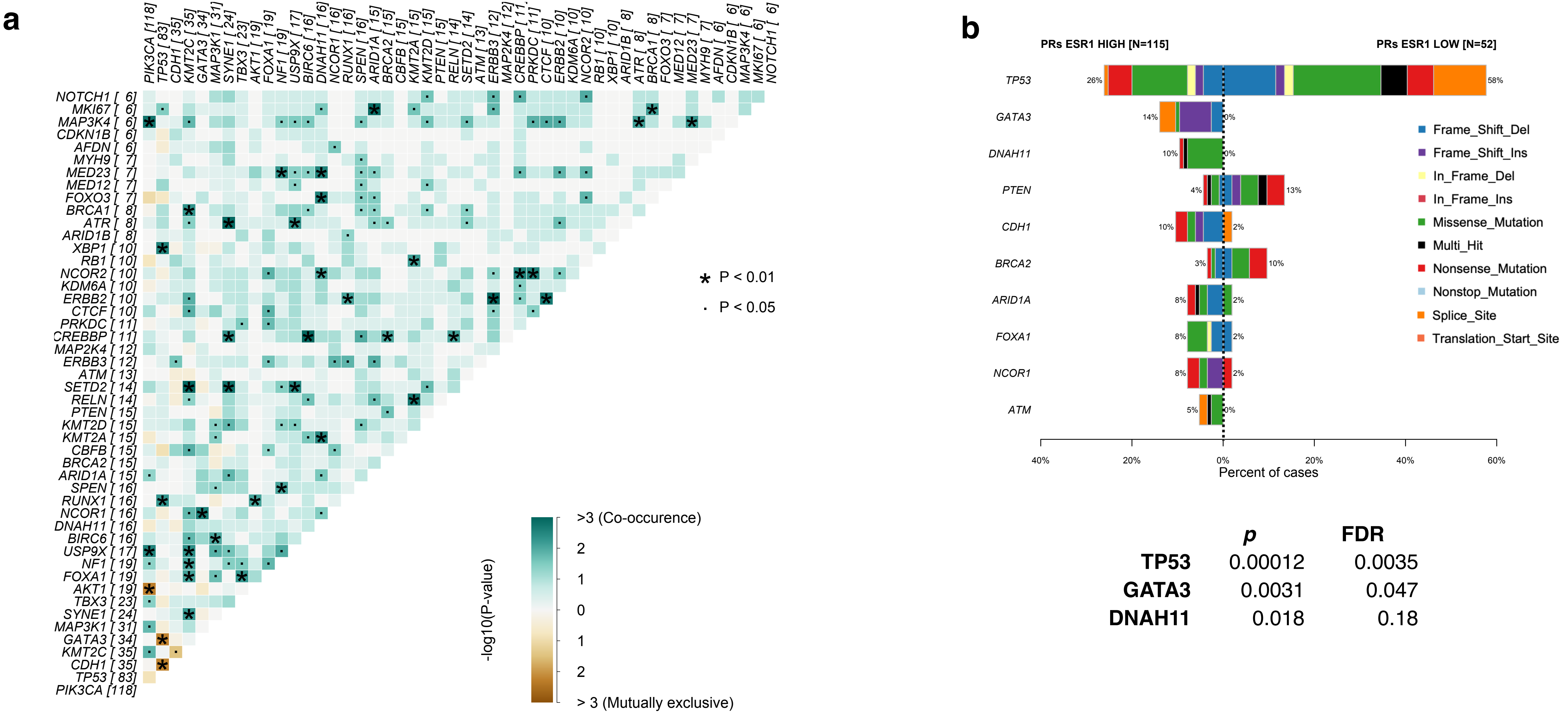

	p	FDR
**TP53**	0.00012	0.0035
**GATA3**	0.0031	0.047
**DNAH11**	0.018	0.18

Supplementary Figure 8. **Somatic mutations in dataset**. **a** Plot showing significance of mutually exclusive or co-occurring set of top mutated genes (>1%) in the full dataset (341 targeted exomes) from the somaticInteractions function in maftools which performs two-sided pair-wise Fisher's Exact test to detect such significant pair of genes. **b** Barplots of top 10 genes and table of significant genes ($p < 0.05$, Fisher's exact two-sided) showing differences between % mutated in PRs ESR1^{HIGH} and PRs ESR1^{LOW}. Ranking of genes based on mafCompare function in maftools. Plot includes variant classifications (frame-shift deletions – blue, frame-shift insertions – purple, missense – green, nonsense – red, nonstop – light blue, splice site – orange, in-frame deletion – yellow, in-frame insertion – dark red, and multi-hit – black). Source data are provided as a Source Data file.

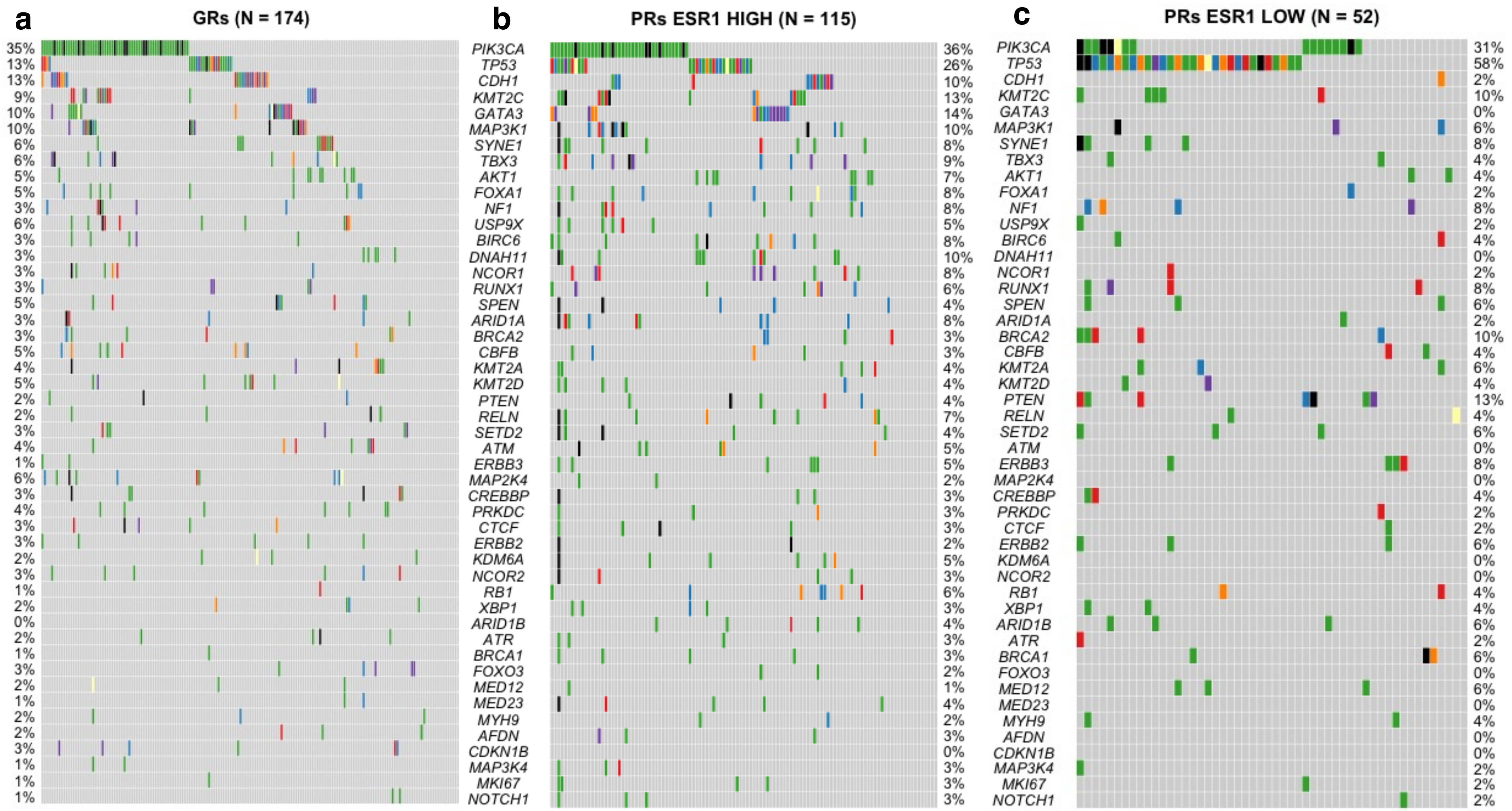

Supplementary Figure 9. **Genes with somatic mutations.** Oncoplot of top mutated genes (>1% in full dataset) for GRs (**a**), PRs ESR1^{HIGH} (**b**) and PRs ESR1^{LOW} (**c**). Plots include variant classifications (frame-shift deletions – blue, frame-shift insertions – purple, missense – green, nonsense – red, nonstop – light blue, splice site – orange, in-frame deletion – yellow, in-frame insertion – dark red, and multi-hit – black). Source data are provided as a Source Data file.

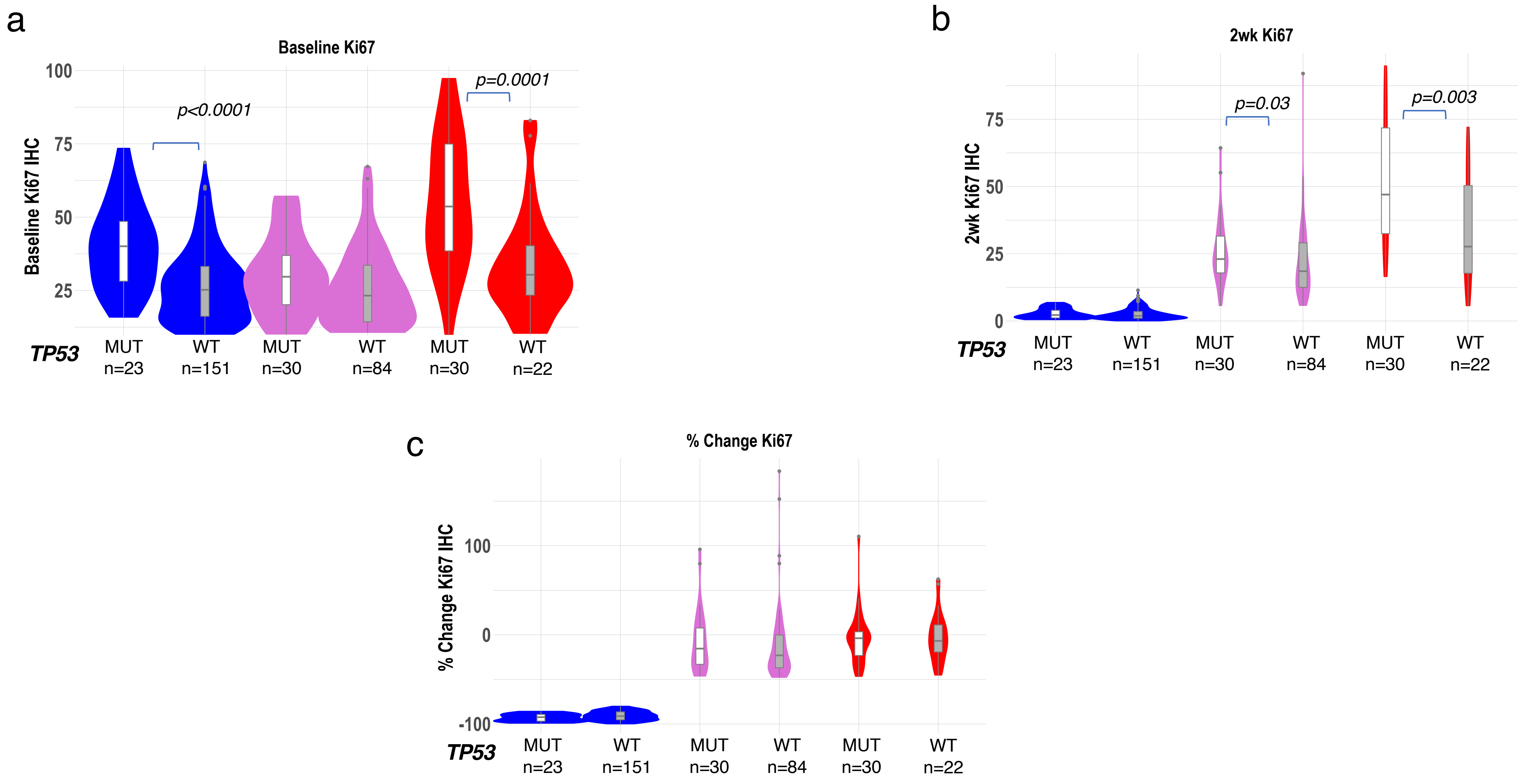

Supplementary Figure 10. **Ki67 and *TP53* mutation status**. Violin/Boxplot of Ki67 at baseline (**a**), Ki67 at 2wks (**b**), and change in Ki67 (**c**) for patients with and without somatic *TP53* mutations. The number of independent samples used for comparisons between GRs and PRs is shown. Boxplots present 25th , 50th (median), and 75th percentile values. Whiskers extend no larger than ±1.5 times the inter-quartile range with outliers plotted individually beyond this range. The number of independent samples used for comparisons between GRs and PRs is shown. Significant differences (p<0.05) determined by two-sided Mann-Whitney tests. Source data are provided as a Source Data file.

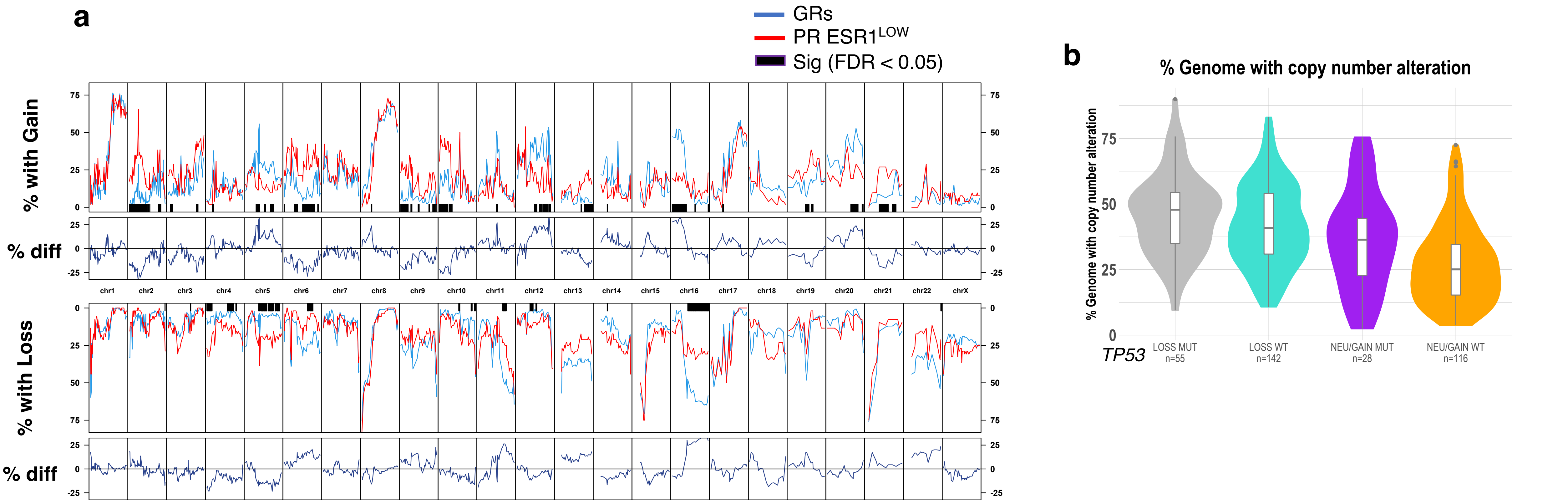

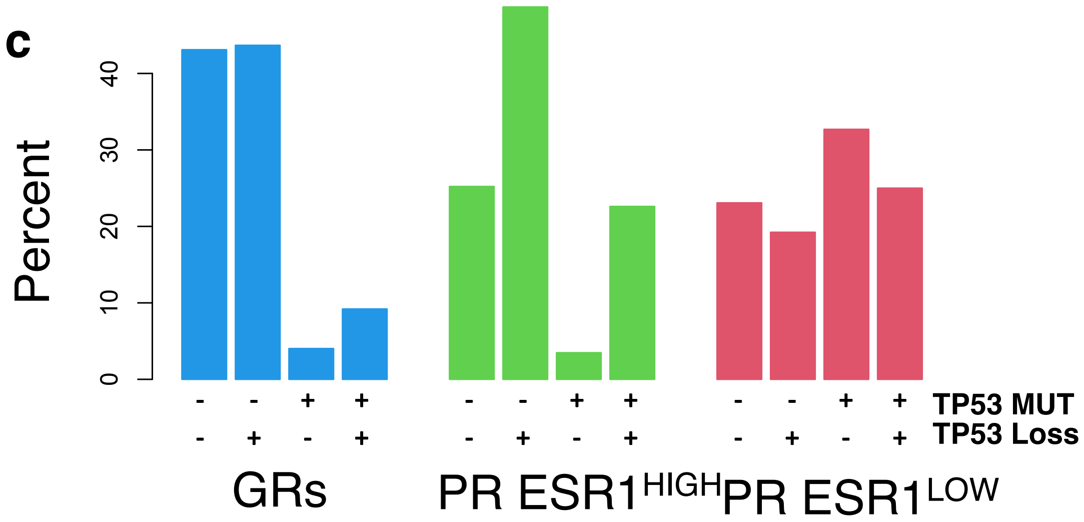

Supplementary Figure 11. **Copy number alterations and TP53 status. a** The percent of GRs (blue) or PRs ESR1^{LOW} (red) with gains or losses at individual chromosomal locations. Black bars highlight regions with significant differences between GRs and PRs ESR1^{LOW} (two-sided fisher-exact test with FDR/Benjamini and Hochberg adjustment). **b** Violin/Boxplot of percent of the genome altered (gains or loss of regions) for tumors with or without loss of *TP53* copy number and/or *TP53* mutations. Boxplots present 25th , 50th (median), and 75th percentile values. Whiskers extend no larger than ±1.5 times the inter-quartile range with outliers plotted individually beyond this range. The number of independent samples used for comparisons between GRs and PRs is shown. **c** Percent of patients with TP53 mutations and/or loss of copy number. Source data are provided as a Source Data file.
