# Supplementary material for: Molecular profiling of aromatase inhibitor sensitive and resistant ER+HER2- postmenopausal breast cancers
Source: Nat Commun. 2023 Jul 7;14:4017. doi: 10.1038/s41467-023-39613-z (PMC10328947; doi:10.1038/s41467-023-39613-z)
Supplement: Supplementary file 3 — Reporting Summary [file 41467_2023_39613_MOESM3_ESM.pdf]

nature portfolio

Corresponding author(s): Eugene F. Schuster

Last updated by author(s): Apr 25, 2023

Reporting Summary

Nature Portfolio wishes to improve the reproducibility of the work that we publish. This form provides structure for consistency and transparency in reporting. For further information on Nature Portfolio policies, see our Editorial Policies and the Editorial Policy Checklist.

Statistics

For all statistical analyses, confirm that the following items are present in the figure legend, table legend, main text, or Methods section.

n/a	Confirmed	
☐	☒	The exact sample size (*n*) for each experimental group/condition, given as a discrete number and unit of measurement
☐	☒	A statement on whether measurements were taken from distinct samples or whether the same sample was measured repeatedly
☐	☒	The statistical test(s) used AND whether they are one- or two-sided<br>*Only common tests should be described solely by name; describe more complex techniques in the Methods section.*
☐	☒	A description of all covariates tested
☐	☒	A description of any assumptions or corrections, such as tests of normality and adjustment for multiple comparisons
☐	☒	A full description of the statistical parameters including central tendency (e.g. means) or other basic estimates (e.g. regression coefficient) AND variation (e.g. standard deviation) or associated estimates of uncertainty (e.g. confidence intervals)
☐	☒	For null hypothesis testing, the test statistic (e.g. *F*, *t*, *r*) with confidence intervals, effect sizes, degrees of freedom and *P* value noted<br>*Give P values as exact values whenever suitable.*
☒	☐	For Bayesian analysis, information on the choice of priors and Markov chain Monte Carlo settings
☒	☐	For hierarchical and complex designs, identification of the appropriate level for tests and full reporting of outcomes
☐	☒	Estimates of effect sizes (e.g. Cohen's *d*, Pearson's *r*), indicating how they were calculated

Our web collection on statistics for biologists contains articles on many of the points above.

Software and code

Policy information about availability of computer code

Data collection

No custom algorithms or software were used in this manuscript. All software with version information are included in manuscript to ensure reproductibility. Citations for software are available in the methods section of manuscript.

RNAseq and targeted exome data were generated on a Illumina Novaseq sequencer

Estradiol data generated on a Waters Acquity® Xevo TQ-XS mass spectrometer

Tumour infiltration was assessed on Nikon H5505 and Nikon E400 microscopes

Chromogenic immunostained slides were scanned using the Aperio AT2 (Leica Biosystems)
RNAseq and targeted exome data were generated on a Illumina Novaseq sequencer

Estradiol data generated on a Waters Acquity® Xevo TQ-XS mass spectrometer

Tumour infiltration was assessed on Nikon H5505 and Nikon E400 microscopes

Chromogenic immunostained slides were scanned using the Aperio AT2 (Leica Biosystems)

Integrative Genomic Viewer version 2.13.0

R version 4.0.2 using Bioconductor version 3.15

CNVkit version 0.9.7

Data analysis

Citations for software are available in the methods section of manuscript.

Analysis of targeted exome DNA:
BWA software (version 0.7.15) was used to align trimmed sequences to genome version hg38. Sequencing adapters were trimmed from fastq files by trim-galore (https://www.bioinformatics.babraham.ac.uk/projects/trim_galore/). Protocols based on GATK version 4.0 to mark read duplicates, recalibrate base quality scores and filter mutation calls were performed on the aligned bam files. Mutect2 from GATK was used to call somatic mutations and Ensembl Variant Effect Predictor was used to annotate the effects of mutations on protein coding genes. Only mutations with 5% allele frequency, observed in at least 5 alternative reads, high to moderate consequences on a protein sequence, and ExAC and gnomAD allele frequencies below 10-5 were included for analysis with maftools. Mutations were visually confirmed using the Integrative Genomic Viewer (https://software.broadinstitute.org/software/igv/).

Analysis of RNAseq:
Salmon was used for quantifying the expression of transcripts based on gencode version 22 GTF transcript annotation. The filterByExpr function in edgeR was used to determine expressed genes and DESeq2 was used for detection of differentially expressed genes. DOSE and cluster profiler were used for gene set enrichment analysis. Consensus Tumor Microenvironment (TME) was used for generating BC specific generating cancer specific signatures for multiple cell types. Single set GSEA (ssGSEA) enrichment scores as calculated in GSVA package in R were used to represent the degree to which the genes within a gene set were coordinately up/down regulated in a sample. Benjamini & Hochberg (1995) method (FDR) was used for multiple correction (p-value adjustment).

Multiplex Immunofluorescence Analysis:
Digital image analysis process took place using QuPath v0.2.3.

For manuscripts utilizing custom algorithms or software that are central to the research but not yet described in published literature, software must be made available to editors and reviewers. We strongly encourage code deposition in a community repository (e.g. GitHub). See the Nature Portfolio guidelines for submitting code & software for further information.

Data

Policy information about availability of data

All manuscripts must include a data availability statement. This statement should provide the following information, where applicable:

- Accession codes, unique identifiers, or web links for publicly available datasets
- A description of any restrictions on data availability
- For clinical datasets or third party data, please ensure that the statement adheres to our policy

The datasets from the POETIC clinical trial are available under restricted access for privacy and legal issues. Access to data or samples can be obtained by submission and approval of a data and sample request form to the POETIC Trial Management Group, which meets several times a year. Data files and details to request access are available from the European Genome-Phenome Archive (EGAD000XXXXXX). The processed data are provided in the Supplementary Information/Source Data files.

Data has been submitted to the EGA but ID has not been generated.

Molecular Signature Database Hallmark gene sets used to calculate the single sample Gene Set Enrichment Analysis (GSEA) can be found in R CRAN package (https://cran.r-project.org/web/packages/msigdbr/index.html) with the following code
msigdbr(species = "Homo sapiens", category = "H")

Breast cancer (BRCA) Consensus Tumor Microenvironment gene sets can be found in the R package ConsensusTME (https://github.com/cansysbio/ConsensusTME)

Human research participants

Policy information about studies involving human research participants and Sex and Gender in Research.

Reporting on sex and gender

Only females were part of the clinical trial. This is a exploratory analysis of a subgroup of the POETIC clinical trial and not the primary analysis of the clinical trial. Details of the trial and primary analysis have been published (https://doi.org/10.1016/S1470-2045(20)30458-7)

Population characteristics

Postmenopausal women with primary estrogen receptor positive Her2 negative breast cancer with palpable or at least 1.5cm tumors. This is a exploratory analysis of a subgroup of the POETIC clinical trial and not the primary analysis of the clinical trial. Details of the trial and primary analysis have been published (https://doi.org/10.1016/S1470-2045(20)30458-7)

Recruitment

4,486 patients were recruited from 130 UK sites over a 5.5 year period. This is a exploratory analysis of a subgroup of the POETIC clinical trial and not the primary analysis of the clinical trial. Details of the trial and primary analysis have been published (https://doi.org/10.1016/S1470-2045(20)30458-7)

Ethics oversight	Patients provided written informed consent before enrolment and POETIC was approved by the London–South East Research Ethics Committee (reference 08/H1102/37)

Note that full information on the approval of the study protocol must also be provided in the manuscript.

Field-specific reporting

Please select the one below that is the best fit for your research. If you are not sure, read the appropriate sections before making your selection.

☒ Life sciences ☐ Behavioural & social sciences ☐ Ecological, evolutionary & environmental sciences

For a reference copy of the document with all sections, see nature.com/documents/nr-reporting-summary-flat.pdf

Life sciences study design

All studies must disclose on these points even when the disclosure is negative.

Sample size	No statistical method was used to predetermine sample size of this substudy of the POETIC trail. Sample size was based on the lowest 15% (n=230) of anti-proliferative response to aromatase inhibitor treatment based on the change in Ki67 between baseline and 2 weeks from all estrogen-receptor positive HER2 negative patients in the POETIC clinical trail (n= 1501). These would roughly represent poor outcome associated tumors with high baseline Ki-67 that remained high after aromatase inhibitor treatment described in Smith, et al. Lancet Oncology 2021. These samples were then matched to tumors the top 50% (n=230) of anti-proliferative response. Matching was based on baseline Ki67 scores. These good responders would roughly represent the moderate risk tumors with high baseline Ki-67 that responded to treatment.
Data exclusions	samples were excluded because RNA/DNA could not be extracted from the tumor, library QC failed and were not sequenced, or alignment coverage was too low (< 20x for targeted exome sequencing). Reasons for excluded included in Supplemental Fig. 1b
Replication	No replication was performed due to the limited amounts of clinical samples available
Randomization	Randomization was not necessary for this analysis as analysis was focused on 2 clearly defined subgroups from the clinical trial (tumors that had good or poor anti-proliferative response to treatment). This is a exploratory analysis of a subgroup of the POETIC clinical trial and not the primary analysis of the clinical trial. Details of the trial randomization have been published (https://doi.org/10.1016/S1470-2045(20)30458-7)
Blinding	Researchers were not blinded to group allocation for this exploratory analysis due to the many factors within the dataset that were previously known and published to be highly correlated with treatment response

Reporting for specific materials, systems and methods

We require information from authors about some types of materials, experimental systems and methods used in many studies. Here, indicate whether each material, system or method listed is relevant to your study. If you are not sure if a list item applies to your research, read the appropriate section before selecting a response.

Materials & experimental systems

n/a	Involved in the study	
☐	☒	Antibodies
☒	☐	Eukaryotic cell lines
☒	☐	Palaeontology and archaeology
☒	☐	Animals and other organisms
☐	☒	Clinical data
☒	☐	Dual use research of concern

Methods

n/a	Involved in the study	
☒	☐	ChIP-seq
☒	☐	Flow cytometry
☒	☐	MRI-based neuroimaging

Antibodies

Antibodies used	Ki67 (MIB1; Cat. M7240; Dako now Agilent)<br>ER (6F11; Cat. NCL-L-ER-6F11; Novocastra)<br>CD3 (LN10; Cat. NCL-L-CD3-565; Novocastra)<br>FOXP3 (236A/E7; Cat. ab20034; Abcam)<br>CD20 (L26; Cat. GA60461-2; Dako)<br>CD68 (514H12; Cat. NCL-L-CD68, Novocastra)
Validation	All antibodies used are commercially available and have been validated by commercial providers.<br><br>Ki67 (MIB1; Cat. M7240; Dako now Agilent) validation - from Agilent website: "In Western blotting of lysates of the multiple myeloma

cell line, IM-9, the MIB-1 antibody labels bands of 345 and 395 kDa, identical to the bands labeled by the original Ki-67 antibody. Furthermore, Western blotting and competitive binding experiments clearly demonstrate that MIB-1, like the original Ki-67 antibody, reacts with an epitope encoded by a 66 bp repetitive element in the Ki-67 gene. In immunohistochemistry, the MIB-1 and the Ki-67 antibodies provide identical staining patterns on serial tonsillar frozen sections (6). The MIB-1 antibody recognizes native Ki-67 antigen and recombinant fragments of the Ki-67 molecule (6)."
References:
1. Gerdes J, Becker MH, Key G, Cattoretti G. Immunohistological detection of tumour growth fraction (Ki-67 antigen) in formalin-fixed and routinely processed tissues. J Pathol 1992,168:85-6.
2. Cattoretti G, Becker MH, Key G, Duchrow M, Schlüter C, Galle J, et al. Monoclonal antibodies against recombinant parts of the Ki-67 antigen (MIB 1 and MIB 3) detect proliferating cells in microwave-processed formalin-fixed paraffin sections. J Pathol 1992,168:357-63.
3. Scholzen T, Gerdes J. The Ki-67 protein: from the known and the unknown [review]. J Cell Physiol 2000,182:311-22.

ER (6F11; Novocastra) validation: https://www.accessdata.fda.gov/cdrh_docs/reviews/K122556.pdf
“Intra-run precision testing of the Estrogen Receptor Clone 6F11 Ready-to-Use Primary Antibody for BondTM (RTU) was evaluated at a single site on FFPE breast cancer tissue on the Bond III staining platform. Similarly the Estrogen Receptor Clone 6F11 Liquid Concentrate Primary Antibody, NovocastraTM (concentrate) was also evaluated at this site on FFPE breast cancer tissue on the Bond III staining platform. The following testing configuration was used in each of the above studies: Testing was conducted using 3 slides mounted with tissue micro arrays (TMAs) of human breast carcinomas samples. Each slide contained 13 TMA cores as follows: 3 cores each of high, medium and low positive ER staining and 3 cores of negative ER staining breast cancer tissue and 1 core of normal breast (non-neoplastic) tissue serving as a control tissue. In addition, 1 test slide with negative control antibody and 1 assay control slide were also used. Staining was performed three times over three different days (3 slides x 3 tests = 9 TMA slides incorporating 108 breast cancer specimens). Two cores were unevaluable due to tissue loss. All test slides were blinded, randomized and assessed by a single observer. The scoring was performed per the ASCO/CAP scoring method [American Society of Clinical Oncology/College of American Pathologists Guideline Recommendations for Immunohistochemical Testing of Estrogen and Progesterone Receptors in Breast Cancer (Arch Pathol Lab Med. 2010;134:e48–e72), i.e. positive for ER if finding of ≥ 1% of tumor cell nuclei are immunoreactive and negative for ER if finding of < 1% of tumor cell nuclei are immunoreactive in the presence of evidence that the sample can express ER (positive intrinsic controls are seen)]. In addition the staining intensity was also assessed. The acceptance criteria set by the sponsor was 85% agreement (overall, positive and negative) at the lower bound of a two-sided 95% confidence interval. The acceptance criteria were met in these studies.”

CD3 (LN10; Novocastra) validation: “Clone LN10 is specific for the non-glycosylated epsilon chain of the human CD3 molecule. Clone LN10 recognizes T cells in thymus, bone marrow, peripheral lymphoid tissue and blood and is a pan T cell marker.”
Reference: Krynitz B, Rozell B and Lindelof B. Acta Dermato Venerologica. 2010; 90:379-385.

FOXP3 (236A/E7; Abcam) validation: Validation in human breast tissue for multiplex immunofluorescence (https://www.abcam.com/products/primary-antibodies/foxp3-antibody-236ae7-bsa-and-azide-free-ab96048.pdf)

CD20 (L26; Dako) validation: Anti-Human CD20cy, clone L26, was clustered as anti-CD20 at the Fifth International Workshop and Conference on Human Leucocyte Differentiation Antigens. SDS-PAGE analysis of immunoprecipitates formed between 125I-labeled tonsil cell lysate and the antibody shows reaction primarily with 30 kDa and 33 kDa polypeptides. Studies using COS-1 cells transfected with cDNA encoding the CD20 molecule, indicate that the antibody labels an intracytoplasmic epitope localized on the CD20 molecule. (https://www.agilent.com/en/product/immunohistochemistry/antibodies-controls/primary-antibodies/cd20cy-%28dako-omnis%29-76218)

CD68 (514H12; Novocastra) validation: Clone 514H12 detected the CD68 glycoprotein in both the cytoplasm and the cell membrane of a variety of cell types of the myelomonocytic cell lineage including monocytes, macrophages, granulocytes, microglial cells, Kupffer cells of the liver, placental Hofbauer cells and a proportion of dendritic cells. (Total number of normal cases evaluated = 47). (https://files.leicabiosystems.com/LBS/GB/en/All?keycode=CD68-L-CE)

Clinical data

Policy information about clinical studies

All manuscripts should comply with the ICMJE guidelines for publication of clinical research and a completed CONSORT checklist must be included with all submissions.

Clinical trial registration: This study is registered with ClinicalTrials.gov, NCT02338310; the European Clinical Trials database, EudraCT2007-003877-21; and the ISRCTN registry, ISRCTN63882543.

Study protocol: This is a exploratory analysis of a subgroup of the POETIC clinical trial and not the primary analysis of the clinical trial. Details of the trial and primary analysis have been published (https://doi.org/10.1016/S1470-2045(20)30458-7)

Data collection: This is a exploratory analysis of a subgroup of the POETIC clinical trial and not the primary analysis of the clinical trial. Details of the trial and primary analysis have been published (https://doi.org/10.1016/S1470-2045(20)30458-7)

Outcomes: This is a exploratory analysis of a subgroup of the POETIC clinical trial and not the primary analysis of the clinical trial. Details of the

Outcomes

trial and primary analysis have been published (https://doi.org/10.1016/S1470-2045(20)30458-7)
