# Supplementary material for: Molecular profiling of aromatase inhibitor sensitive and resistant ER+HER2- postmenopausal breast cancers
Source: Nat Commun. 2023 Jul 7;14:4017. doi: 10.1038/s41467-023-39613-z (PMC10328947; doi:10.1038/s41467-023-39613-z)
Supplement: Supplementary file 4 — Description of Additional Supplementary Files [file 41467_2023_39613_MOESM4_ESM.pdf]

Description of Additional Supplementary Files

File name: Supplementary Data 1

Description: Excel worksheets with differential gene expression analysis from DESeq2 for comparisons GRs vs all PRs, GRs vs PRs $ESR1^{HIGH}$, GRs vs PRs $ESR1^{LOW}$, and PRs $ESR1^{HIGH}$ vs PRs $ESR1^{LOW}$.

File name: Supplementary Data 2

Description: Excel worksheets with enriched Gene Ontology Biological Processes and MSD Hallmarks for genes up or down regulated (FDR < 0.05) for comparisons GRs vs PRs $ESR1^{HIGH}$, GRs vs PRs $ESR1^{LOW}$.

File name: Supplementary Data 3

Description: Excel worksheets with copy number analysis using Fisher-exact test to determine differences between the number of GRs and PRs ($ESR1^{HIGH}$ and $ESR1^{LOW}$) for cytoband regions.

File name: Supplementary Data 4

Description: Excel worksheet for location of targeted regions for exome sequencing.
